# Solution-processed Cu_2_ZnSnS_4_ thin film with mixed solvent and its application in superstrate structure solar cells†

**DOI:** 10.1039/c8ra01095a

**Published:** 2018-03-22

**Authors:** Rongjing Yan, Li Kang, Yuxiu Sun, Jingbo Zhang

**Affiliations:** Key Laboratory of Inorganic-Organic Hybrid Functional Material Chemistry, Ministry of Education, Tianjin Key Laboratory of Structure and Performance for Functional Molecules, College of Chemistry, Tianjin Normal University Tianjin 300387 China hxxyzjb@mail.tjnu.edu.cn

## Abstract

Cu_2_ZnSnS_4_ (CZTS) thin film solar cells become an interesting research topic due to some advantages of the CZTS thin film such as having nontoxic and abundant components, a low price and excellent optoelectronic properties. In this work, a solution-based preparation method was developed to fabricate a CZTS solar cell with a superstrate structure of FTO/TiO_2_/CdS/CZTS/P3HT/Cu by using mixed solvent. Nanocrystalline TiO_2_ porous thin film was used as the bottom layer for deposition of CZTS to increase the interfacial area of CZTS. To deposit CZTS inside the porous structure leading to a good contact of CZTS with porous TiO_2_ thin film, the CZTS precursor particle size is successfully regulated by changing the volume ratios of *N*,*N*-dimethylformamide and ethanol. More importantly, small size CZTS precursor particles can easily enter into the porous structure of nanocrystalline thin film leading to a good interfacial contact, which allowed the effective improvement of the light-to-electric conversion efficiency for the present superstrate CZTS solar cell. This work may provide a promising way for the design of high-efficient superstrate solar cells.

## Abstract 2

Solution processed superstrate CZTS solar cells with mixed solvent to adjust CZTS particle size.

## Graphical abstract

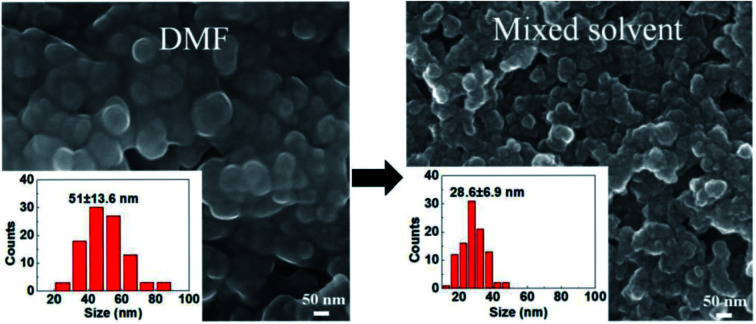

## 1. Introduction

Recently, the quaternary Cu_2_ZnSnS_4_ (CZTS) as a potential substitute for CuInGaSe_2_ (CIGS) has become a photovoltaic application potential candidate.^1^ The structure of CZTS is similar to that of CIGS, and the bandgap of the CZTS (approximate 1.5 eV) is well matched with the solar spectrum.^2^ In addition, the light absorption coefficient is high in the visible light region (10^4^ cm^−1^). Researchers began to explore CZTS, with similar capabilities to CIGS, because of its excellent light absorption properties and a wealth of elements. As a result, the photoelectric conversion efficiency of CZTS (Se) solar cells was increased from 0.66% in 1996 to 6.7% in 2008, and then to 12.6% in 2012.^3^ There is no doubt that the CZTS film will soon become another promising material, as the preparation technology has been continuously improved.

Most CZTS photovoltaic devices are fabricated with a configuration of Mo/CZTS/buffer/metal oxide nanostructures/metal electrodes,^4^ where the p-type semiconductor absorber layer directly contacts with the molybdenum back prepared by sputtering, spray pyrolysis, electrodeposition, thermal evaporation, *etc.* Previous reports have shown that this configuration has some disadvantages such as deterioration of the metal oxide due to the thermal instability of the absorber layer leading to formation of the buffer interface.^5^ In addition to these limitations, in CZTS solar cells there are the erosion reaction between CZTS and the Mo back contact and the surface instability of CZTS during the heat treatment.^6^ These problems can be ameliorated by the introduction of a superstrate structure in solar cell, where metal oxide is deposited on a smooth top substrate and then annealed at high temperature to prevent direct contact of CZTS and the conducting substrate.^7^ In previous work, the CZTS growth on TiO_2_ thin films offered the highest conversion efficiency of 0.25%.^8,9^ Most of following works are to change the morphology of metal oxides to improve the conversion efficiency of CZTS solar cells.^10^ There are also some of works focusing on using non-toxic buffer material^11^ or replacing the counter electrode^4^ to achieve the desired efficiency of solar cells. The reported conversion efficiency of the superstrate structure was gradually increased from 0.84% to 3.63%.^12–17^ The superstrate structure is beneficial in supplying enough driving force to facilitate electron injection,^18^ the CZTS absorber-TiO_2_ conductor interface plays a crucial role in the device's efficiency, however, there is few study to discuss this issue.

In 2013, Wang *et al.* found the main reason for the low conversion efficiency of CZTS solar cell is that CZTS is only distributed on the surface of TiO_2_ nanoparticles and does not fill in the voids of the film like dye molecules.^19^ In the same year, Li *et al.* pointed out that smaller size and more evenly distributed CZTS nanoparticles are expected to achieve a higher conversion efficiency of CZTS solar cell.^20^ It is well-known that the performance of thin film solar cells depends greatly on the morphology of the absorber layer. Therefore, good absorber layer morphology is the key to improve the conversion efficiency of solar cell. There are several ways to obtain different morphologies. It is important to choose a solvent. Here, we mainly explore the impact of the mixed solvent on the morphology of CZTS. On the one hand, good solvents should have high solubility for the metal source and the sulfur source, and low surface tension to make the solution be easy to completely cover without streaks or voids. On the other hand, they should have proper boiling point and vapor pressure allowing the solvent to be evaporated easily, and have minimal residue leading to microstructure stability during a rapid volatilization process. In some fields, DMF is a common solvent that meets the above requirements.^21^ Here, in order to improve the coverage of CZTS on TiO_2_ nanoparticles, we tried to add ethanol in DMF solvent for preparation of CZTS precursor solution, since solvent surface tension has different effects on CZTS growth processes. This treatment can improve the TiO_2_ surface wettability and make better contact interface. We hope that the addition of ethanol can make the CZTS precursor solution enter into the porous TiO_2_ film better and improve the photoelectric conversion efficiency of superstrate structure solar cell. Therefore, we explored the effect of the mixed solvent ratio on the morphology of CZTS precursor. The size of CZTS particles significantly decreases with the increase of ethanol with the lower surface tension. The prepared TiO_2_/CZTS thin film was used as a photoelectrode to fabricate the superstrate CZTS thin film solar cell. The highest light-to-electric conversion efficiency for a superstrate structure CZTS solar cell was achieved to 2.15%.

## 2. Experimental

### 2.1 Materials

Titanium chloride (TiCl_4_), titanium dioxide (TiO_2_) P25 nanoparticle powders, potassium chloride (KCl), copper chloride (CuCl_2_·2H_2_O), zinc chloride, anhydrous (ZnCl_2_), tin chloride (SnCl_2_), thiourea (CH_4_N_2_S), cadmium sulfate (CdSO_4_), ammonia (NH_3_·H_2_O) and P3HT were purchased from Alfa Aesar Inc. China. FTO conducting glass (10 Ω sq^−1^) was purchased from Nippon Sheet Glass Co., Ltd. Japan.

### 2.2 Preparation of porous thin films

FTO conducting glass substrate was ultrasonically cleaned using acetone, ethanol and deionized water. P25 powders were used to prepare nanocrystalline TiO_2_ thin film by the doctor-blade method.^22^ Then a thin layer of ZnO was electrodeposited to passivate the surface defects of TiO_2_ according to the reported method.^23^ Further, a layer of CdS was deposited by the chemical bath deposition.^24^ The obtained FTO/nano-TiO_2_/ZnO/CdS thin film was used as a substrate for further deposition of CZTS.

### 2.3 Solution deposition of CZTS

The precursor solution consists of 0.12 M CuCl_2_·2H_2_O, 0.07 M ZnCl_2_, 0.08 M SnCl_2_ and 0.42 M thiourea in DMF or the mixed solvent of DMF and ethanol. Four compounds are added in the order of CuCl_2_, SnCl_2_, ZnCl_2_, and thiourea. The above solution was stirred for 5 min. CZTS precursor thin film was prepared *via* direct solution spin coating at 3000 rpm for 20 s and then dried in air at 150 °C for 2 min to remove the excess solvent and organic residues. This process was repeated twice to give a uniform coating film with the desired thickness. The as-prepared thin film was annealed at 550 °C for 60 min under sulfur atmosphere. Then, the sample was allowed to be cooled naturally to room temperature under an inert atmosphere. P3HT as the hole transport materials were deposited on the surface of CZTS by the spin coating method, and the Cu electrode was further deposited using a vacuum evaporation plating instrument (VZZ-300) to form a CZTS solar cell with the superstrate structure of FTO/nano-TiO_2_/ZnO/CdS/CZTS/P3HT/Cu.

### 2.4 Characterization of CZTS thin films

The morphologies of the CZTS on nanoporous TiO_2_ thin films were observed using a scanning electron microscope (SEM, JSM-7500F, JEOL). The composition of the films were analyzed by energy dispersive spectroscopy (EDS) attached to the SEM. The crystalline structure of the samples was characterized by an X-ray power diffraction meter (XRD, RigakuD/max-2500, Cu Kα). Raman spectra were obtained with Horiba Labram HR Evolution, the laser wavelength for Raman spectrum is 532 nm. X-ray photoelectron spectroscopy (XPS) measurements were performed using an AXIS ULTRA of Al Kα (1486.6 eV) X-ray source. The optical properties of the CZTS films were studied using a UV-2600 spectrophotometer at room temperature. *J*–*V* curves of the superstrate CZTS solar cells were measured under illumination with a solar simulator (Newport 94023A) at 1 sun (AM1.5, 100 mW cm^−2^). A potentiostat (Solartron 1287/1260) was used to control the potential and record the current values.

## 3. Results and discussion

### 3.1 The effect of mixed solvent on morphologies of CZTS thin film

**Fig. 1:**
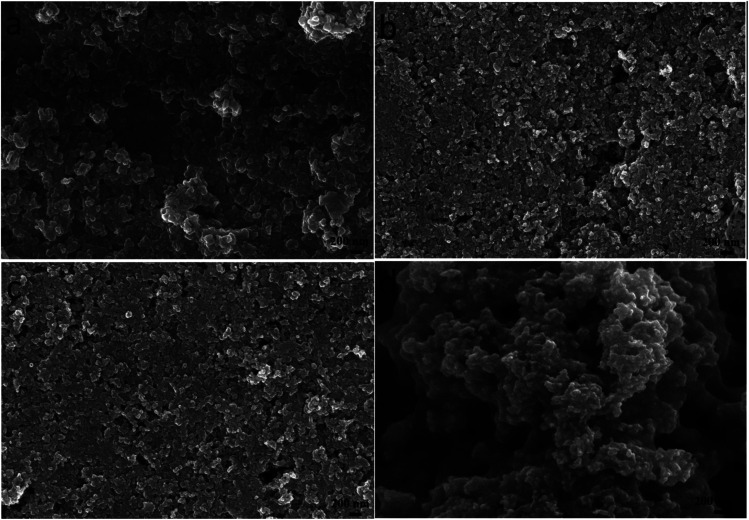
Surface SEM images of CZTS thin films prepared from precursors in DMF (a), DMF : ethanol = 9 : 1 (b), DMF : ethanol = 1 : 1 (c) and ethanol (d).

Due to the different solvent evaporation rate and the solubility of different materials, the surface morphology of CZTS precursor films varies obviously. As depositing CZTS on the nanocrystalline TiO_2_ thin film, the particle size of CZTS should be small so that they can enter into the porous structure fully, namely, CZTS can wholly fill in the porous structure to make a well contact interface between CZTS and nanocrystalline TiO_2_. Therefore, we want to carefully control the size of CZTS particles by adjusting the mixed solvent ratio. The surface morphologies of the films prepared through CZTS precursor solutions with different solvents were showed in Fig. 1. It can be seen that there are many bigger particles forming a pore structure with some cracks on the surface of the film prepared from CZTS precursor solution in DMF as shown in Fig. 1a. These cracks will lead to the worse performance of CZTS solar cell. In order to effectively eliminate these cracks, the precursor solution is mixed with a small molecule solvent such as ethanol. As can be seen from Fig. 1b, after the addition of 10% (volume percent) ethanol into DMF as the mixed solvent, the nanoparticles are uniformly distributed, and the big pores are almost absent. The SEM image clearly shows that CZTS thin film has a flat surface. It is obvious that the mixed solvent in the precursor solution can change the porosity of the final thin film. As 50% ethanol was mixed with DMF, the obtained thin film shows more flat surface as shown in Fig. 1c. However, compared to pure DMF, pure ethanol was used as solvent to prepare CZTS thin film, due to fast solvent evaporation, there is agglomeration of small particles and it becomes very rough with many bigger holes (Fig. 1d).

**Fig. 2:**
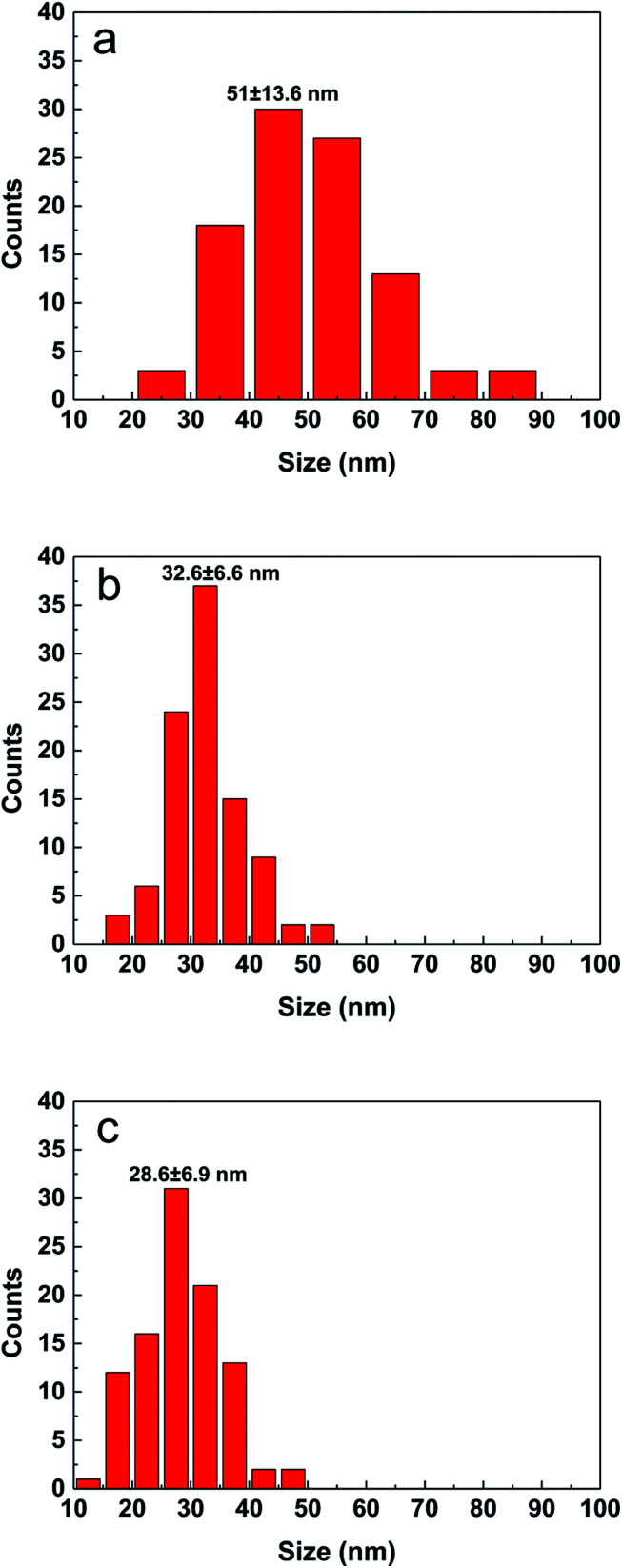
Particle size distribution of CZTS particles prepared from precursors in DMF (a), DMF : ethanol = 9 : 1 (b) and DMF : ethanol = 1 : 1 (c).

By adjusting the reactivity of the precursor and the coordination ability of the solvent, the average particle size of the CZTS nanocrystals is in the range of 10 to 60 nm in different cases as seen from Fig. 1S.† The shape of nanoparticles is spherical. The film prepared with the DMF solution consists of inhomogeneous particles. The aggregation of difference size particles leads to some defects and cracks, which will degrade the performance of solar cell. The particle size distribution was estimated from SEM surface images by counting more than 100 particles and shown in Fig. 2. When the precursor solution is mixed with a small molecule ethanol, the average particle size is decreased from 51.0 nm (DMF) to 32.6 nm for the sample made from the mixed solution with the volume ratio of 9 : 1 (DMF : ethanol). And the average particle size was further decreased to 28.6 nm in the case of their volume ratio of 1 : 1. Due to the addition of ethanol, the distribution becomes more narrow meaning the particles become more homogeneous in size.

**Fig. 3:**
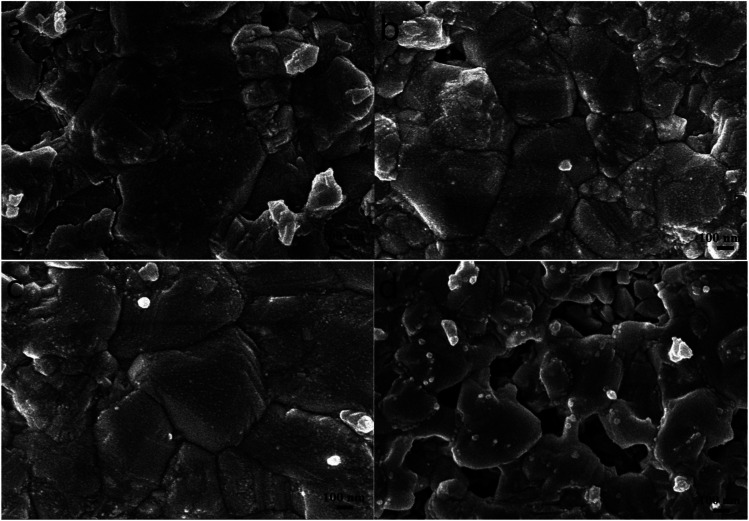
Surface SEM images of the sulfurized CZTS prepared from precursors in DMF (a), DMF : ethanol = 9 : 1 (b), DMF : ethanol = 1 : 1 (c) and ethanol (d).

In order to promote crystallization of CZTS on the surface of TiO_2_ nanoparticles, the precursor film was further subjected to a sulfurization treatment at 550 °C for 60 min. Fig. 3 shows top-view SEM images of the typical CZTS thin films. The CZTS morphologies on nanoporous TiO_2_ show a sharp change compared with the film deposited directly on the flat substrate.^21^ It is observed from the SEM image that the film is dominated by several large grains. As seen from Fig. 3a, they are relatively dense with a majority of grains bigger than 0.5 μm, and a small part of grains less than 0.2 μm. In addition, the addition of ethanol solvent makes the film appear more uniform and smooth as shown in Fig. 3b and c. However, using ethanol as the solvent, the grain growth was not complete after the sulfurization treatment, the number of holes becomes more and the surface is not flat (Fig. 3d).

**Fig. 4:**
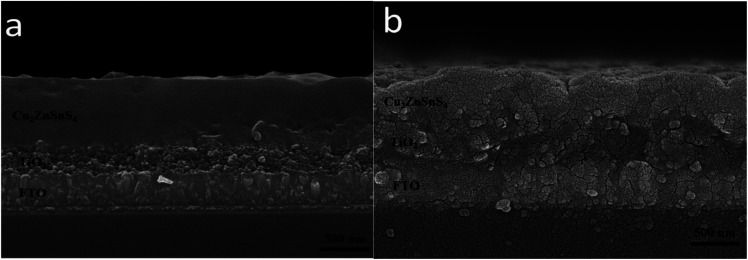
SEM cross section images of the films prepared from DMF : ethanol = 1 : 1 solvent before (a) and after (b) sulfurization.

The cross-sectional SEM images of the CZTS film on TiO_2_ nanocrystalline thin film prepared with the mixed solvent of DMF and ethanol with a volume ratio of 1 : 1 before and after sulfurization were shown in Fig. 4. Surface of the film before sulfurization is smooth and homogeneous, no significant cracks or voids were observed as shown in Fig. 4a. The interface between CZTS and nanocrystalline TiO_2_ is not clear meaning CZTS particles enter into porous structure of nanocrystalline thin film. The film thickness is approximately 600 nm including the TiO_2_ layer of about 200 nm thick and the CZTS layer of about 400 nm. After the sulfurization treatment, the grains become larger and extended through the CZTS layer, the thickness is almost unchanged as shown in Fig. 4b.

### 3.2 The effect of mixed solvent on composition and structure of CZTS thin film

**Fig. 5:**
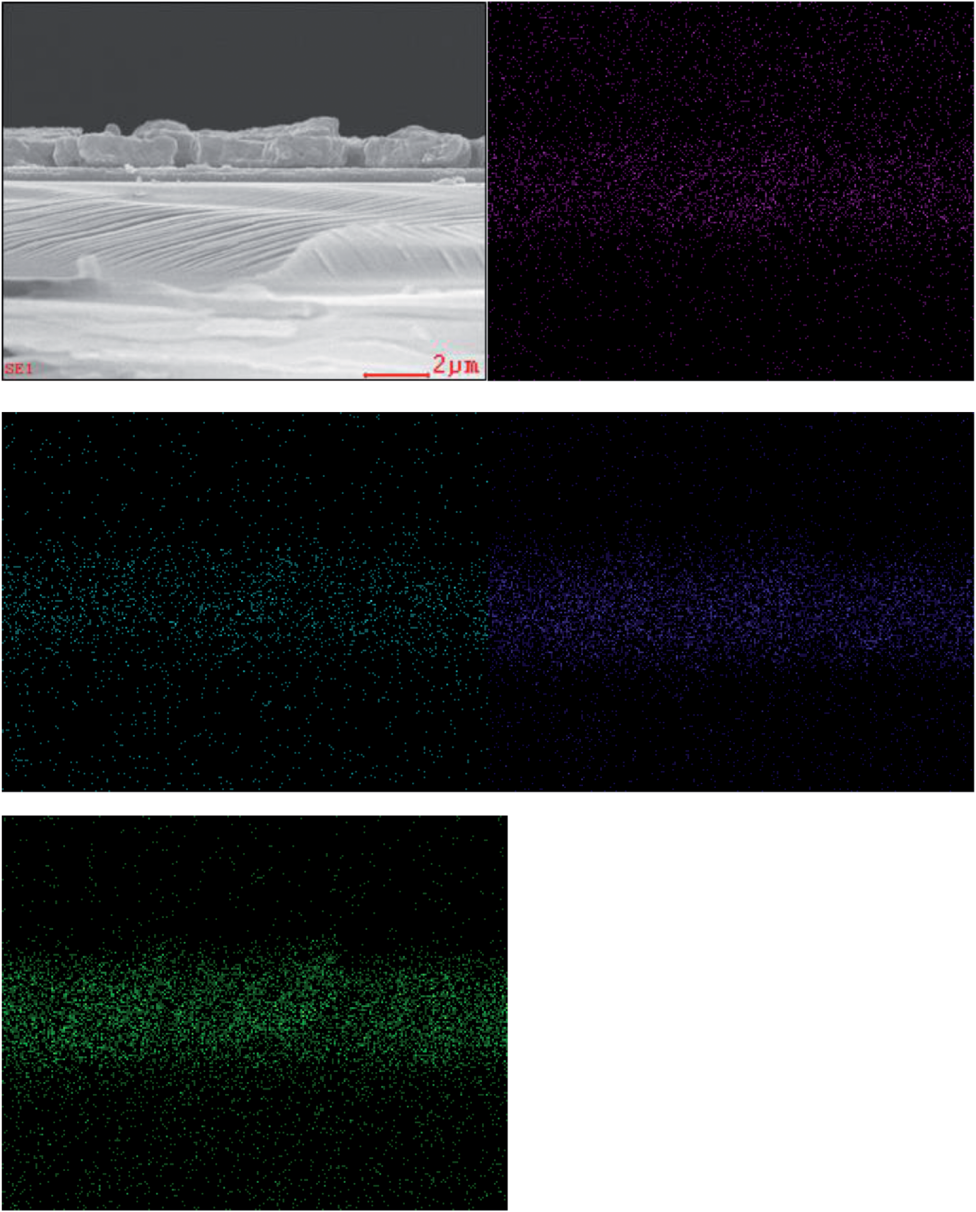
Cross section EDS color map of the CZTS thin film prepared from precursors in DMF : ethanol = 1 : 1 solvent. Cu, Zn, Sn and S are represented with purple, cyan, blue and green, respectively.

Composition is a key parameter to determine the formation of the quaternary CZTS compound. The elemental distribution of the CZTS thin film prepared by the mixed solvent of 1 : 1 was identified by elemental EDS analyses. The cross section EDS color map was shown in Fig. 5. In the map, green, blue, cyan and purple represent S, Sn, Zn and Cu, respectively. The EDS analyses of the sample showed that the constituent elements Cu, Zn Sn and S were present in the sample and distributed almost uniformly on the whole layer. Meanwhile, the corresponding average atomic percentage of Ti, O, Cu, Zn, Sn and S were obtained (Fig. 2S and Table 1S†). And the atomic ratio percentage of Cu : Zn : Sn : S is closed to 2 : 1 : 1 : 4. The bandgap of the prepared CZTS thin film was estimated to be 1.5 eV from the absorption spectrum (Fig. 3S†).

**Fig. 6:**
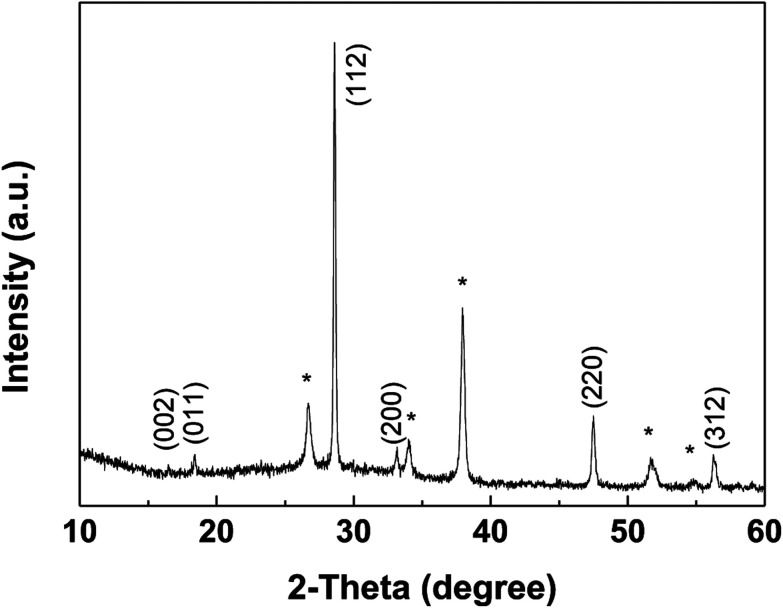
XRD patterns of the CZTS thin film prepared from precursors in DMF : ethanol = 1 : 1 solvent, the diffraction peaks of FTO were marked with *.

The XRD pattern of the CZTS thin film prepared with the mixed solvent of DMF and ethanol with a volume ratio of 1 : 1 after annealed at 550 °C for 60 min in sulfur atmosphere was shown in Fig. 6. Sample shows three major diffraction peaks at 28.6°, 47.6° and 56.3°, which can be indexed to the (112), (220) and (312) in the kesterite structure of Cu_2_ZnSnS_4_ (JCPDS no. 26-0575), respectively. No noticeable impurities can be observed. The lattice parameters, *a* = *b* = 5.41 Å, *c* = 10.8 Å, calculated from the pattern, are in good agreement with the standard values for tetragonal CZTS.^25^

**Fig. 7:**
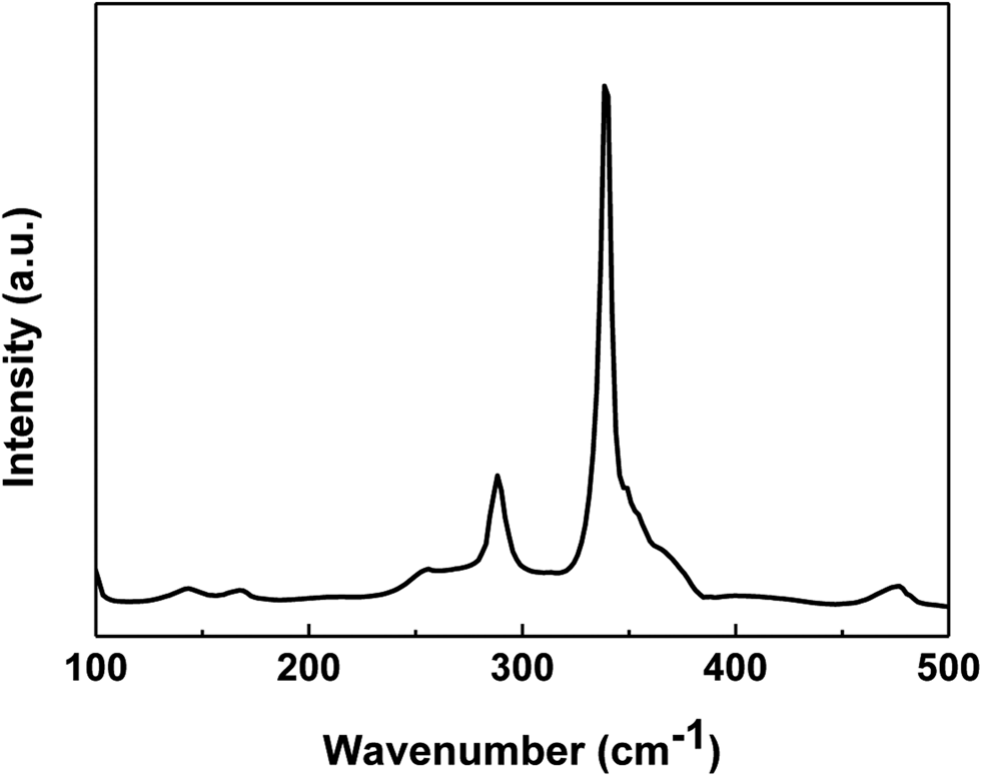
Raman spectrum of the CZTS thin film prepared from precursors in DMF : ethanol = 1 : 1 solvent.

The XRD pattern is not enough to identify the phase purity of the synthesized product because some binary (cubic ZnS) and ternary (tetragonal Cu_2_SnS_3_) chalcogenides have similar lattice parameters or pattern characteristics to CZTS. Thus, Raman spectrum on the CZTS surface was measured to detect the presence of the secondary phase. Fig. 7 shows the Raman spectrum of the CZTS thin film prepared with the mixed solvent of DMF and ethanol with a volume ratio of 1 : 1. The major peaks at 338 cm^−1^ and 286 cm^−1^ are attributed to the CZTS A1 mode. The A1 phonon mode is pure anion mode, which puts to the vibration of S atoms surrounded by motionless neighboring atoms.^26^ Other possible binary phases such as ZnS, Sn_2_S_3_ and Cu_*x*_S for the obtained CZTS thin film cannot be excluded based on the Raman spectrum.^27^

**Fig. 8:**
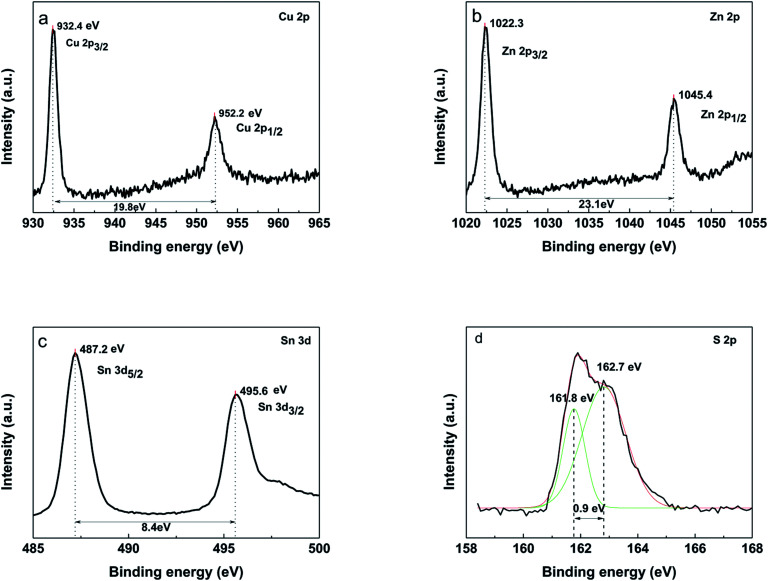
XPS of the CZTS thin film prepared from precursors in DMF : ethanol = 1 : 1 solvent, (a) Cu 2p, (b) Zn 2p, (c) Sn 3d, (d) S 2p.

X-ray photoelectron spectroscopy (XPS) was used to further confirm the presence of four components (Fig. 8). The peaks at 932.4 eV and 952.2 eV on Cu 2p spectra (Fig. 8a) correspond to 2p_3/2_ and 2p_1/2_ (generated by spin orbital splitting), respectively. Two peaks are divided into 19.8 eV. No satellites are found in the higher binding direction of the peak. Thus, it can be concluded that only Cu^+^ is present in the sample, indicating that Cu^2+^ was reduced during this process. For the Zn 2p spectrum, between 2p_3/2_ and 2p_1/2_, there is a spin orbital division of 23.1 eV (Fig. 8b). The peaks of Zn 2p appear at 1022.3 eV and 1045.4 eV, being consistent with the standard distribution of Zn^2+^. The two lines corresponding to Sn of 3d_5/2_ and 3d_3/2_ were observed at 487.2 eV and 495.6 eV, respectively. Sn^4+^ was confirmed by a peak resolution of 8.4 eV (Fig. 8c). It indicates that Sn^2+^ in the starting material is oxidized to Sn^4+^. The analysis of the sulfur core state of the annealed CZTS thin film (Fig. 8d) indicates the peaks at 161.8 eV and 162.7 eV are corresponding to the S 2p_3/2_ and S 2p_1/2_.^28,29^

### 3.3 The effect of mixed solvent on performance of superstrate CZTS solar cells

**Fig. 9:**
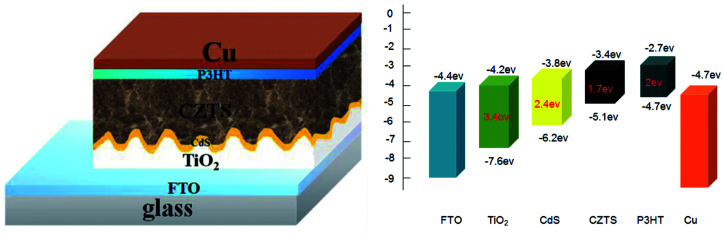
Schematic diagram of superstrate FTO/TiO_2_/CdS/CZTS/P3HT/Cu solar cell and the corresponding bandgap alignment of each layer.

**Fig. 10:**
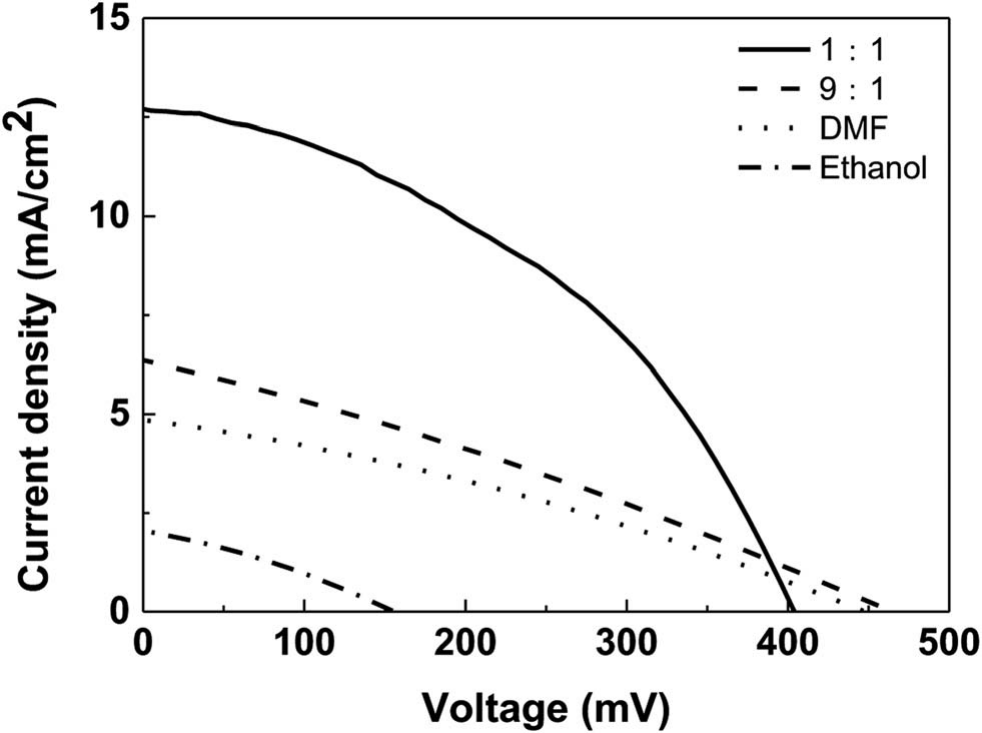
*J*–*V* curves of CZTS solar cells based on CZTS thin films prepared from precursors in different solvents.

To fabricate a superstrate CZTS solar cell, P3HT was spin-coated to form a hole transport layer on the prepared FTO/TiO_2_/CdS/CZTS thin film, and then the copper electrode was further vacuum-evaporated. The schematic superstrate structure and the typical band diagram of CZTS solar cell was shown in Fig. 9. P3HT hole transport layer is necessary, which can obviously improve the photovoltaic performance (Fig. 4S†). The *J*–*V* curves of the as-fabricated CZTS solar cells were measured and shown in Fig. 10.

Unfortunately, the performance of the superstrate CZTS solar cell is poor with a short-circuit photocurrent density (*J*_sc_) of 3.5 mA cm^−2^. The low photocurrent density is usually due to presence of interfacial states as recombination sites and chemical composition of CZTS. TiCl_4_ hydrolysis was usually used to form a dense TiO_2_ layer to modify these recombination sites. However, the conversion enhancement is not obvious. Alternately, a thin layer of ZnO was electrodeposited on the porous surface of nanocrystalline TiO_2_ thin film. One of advantages for electrodeposition is the uniform layer can be formed on a rough electrode surface. Due to porous structure of nanocrystalline TiO_2_ thin film, the electrodeposition of ZnO could better modify surface defects and improve conversion efficiency of the superstrate solar cell. Firstly, the thickness of the electrodepositing ZnO layer was optimized (Fig. 5S†), the photovoltaic parameters of CZTS solar cells with the ZnO layer deposited for different times were collected in Table 1. Electrodeposition of ZnO layer for 0.5 min shows the best passivation effect towards the recombination sites and gives highest conversion efficiency.

**Table 1:** Photovoltaic parameters of CZTS solar cells with the ZnO layers electrodeposited for different times

Electrodeposition time (min)	*J*_sc_ (mA cm^−2^)	*V*_oc_ (mV)	ff (%)	*η* (%)
0	3.5	430	34.1	0.51
0.3	4.5	450	30.9	0.63
0.5	12.6	400	42.3	2.15
0.7	6.1	460	34.5	0.97

Further, the effect of mixed solvent with different volume ratios on the performance of superstrate CZTS solar cell was studied and *J*–*V* curves corresponding to different solar cells were shown in Fig. 10. *J*_sc_ values of solar cells with the CZTS thin films prepared in DMF solvent, mixed solvents with volume ratios of 9 : 1 and 1 : 1 were 4.9 mA cm^−2^, 6.4 mA cm^−2^ and 12.6 mA cm^−2^, respectively. The increase in *J*_sc_ indicates that the electron–hole recombination rate in the 1 : 1 case is lower than that of other two samples. This may be due to the fact that the formed small CZTS particles enter into porous structure forming a well interfacial contact with nano-TiO_2_/CdS layer, which contributes to the rapid transfer of electrons. Therefore, the solar cell based on CZTS prepared by the 1 : 1 mixed solvent has the highest energy conversion efficiency (*η*) of 2.15%, followed by the solar cells based on the 9 : 1 solvent (0.86%) and DMF (0.68%). However, only ethanol was as solvent to prepare CZTS, there is no expected effect, the conversion efficiency is only 0.10%.

## 4. Conclusions

A pure DMF solvent, pure ethanol solvent and DMF/ethanol mixed solvents with different proportions was used to develop a direct solution method to prepare CZTS thin film on nanocrystalline TiO_2_ thin film modified by CdS. The effects of different ratios of mixed DMF/ethanol solvent on solar cell performance were compared. The results showed that the crystallinity of CZTS was better with the proper proportion of DMF/ethanol mixture. The volume ratio increasing of ethanol in the mixed solvent leads to formation of smaller CZTS particles. For the nanoporous thin film, smaller particles can well enter inside the porous structure and make a good interfacial contact. FTO/nano-TiO_2_/CdS/CZTS was used as a photoelectrode to fabricate the superstrate solar cell with the P3HT layer as hole transport material and Cu as the counter electrode. To modify the interfacial sites presenting on the surface of TiO_2_ nanocrystals, a thin layer of ZnO was electrodeposited. Electrodeposition of 0.5 min shows the best modification and gives the light-to-electric conversion efficiency of 2.15%.

## Conflicts of interest

There are no conflicts to declare.

## Supplementary Material

RA-008-C8RA01095A-s001
