# Supplementary material for: Solution-processed Cu2ZnSnS4 thin film with mixed solvent and its application in superstrate structure solar cells
Source: RSC Adv. 2018 Mar 22;8(21):11469–77. doi: 10.1039/c8ra01095a (PMC9079141; doi:10.1039/c8ra01095a)
Supplement: RA-008-C8RA01095A-s001 [file RA-008-C8RA01095A-s001.pdf]

Electronic Supplementary Material (ESI) for RSC Advances.
This journal is © The Royal Society of Chemistry 2018

Solution-processed Cu_2ZnSnS_4 thin film with mixed solvent and its application in superstrate structure solar cell

Rongjing Yan, Li Kang, Yuxiu Sun* and Jingbo Zhang*

Key Laboratory of Inorganic-Organic Hybrid Functional Material Chemistry, Ministry of Education, Tianjin Key Laboratory of Structure and Performance for Functional Molecules, College of Chemistry, Tianjin Normal University, Tianjin 300387, China

* Corresponding authors.

Jing Bo Zhang

College of Chemistry, Tianjin Normal University, Tianjin 300387, China

hxxyzjb@mail.tjnu.edu.cn

Supporting Information

Table.1S Summary of EDAS analysis performed in CZTS prepared by the mixing solvent with 1:1 volume ratio of DMF and ethonal.

Elements	O	S	Ti	Cu	Zn	Sn	Total
Percent of weigh (%)	47.11	3.04	37.40	4.73	2.31	5.40	100
percent of atom (%)	74.07	2.39	19.64	1.87	0.89	1.14	100

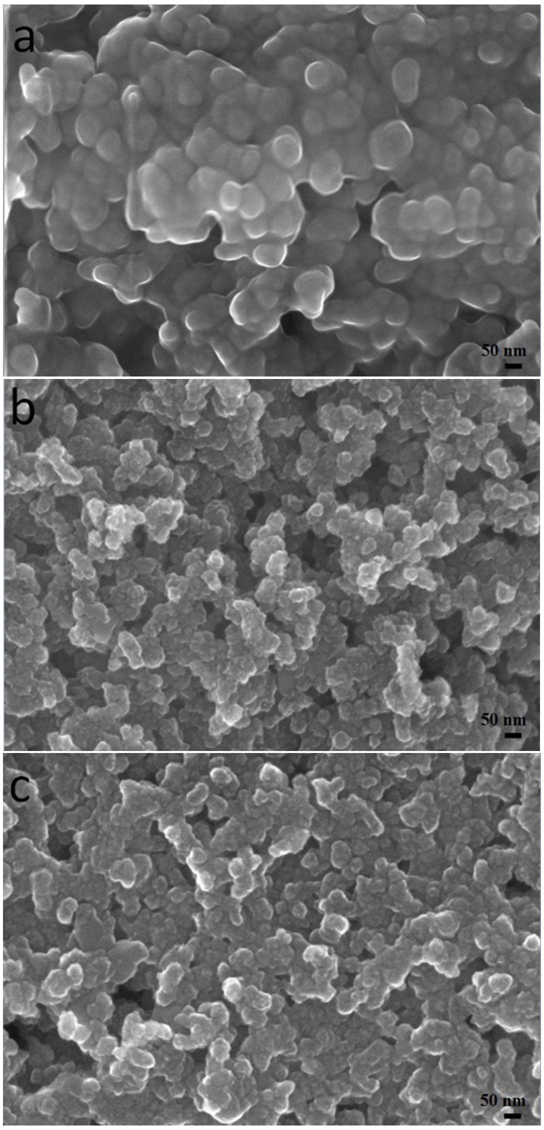

Fig.1S Surface SEM images of CZTS thin films prepared from precursors in DMF (a), DMF:Ethanol=9:1 (b), DMF:Ethanol=1:1 (c).

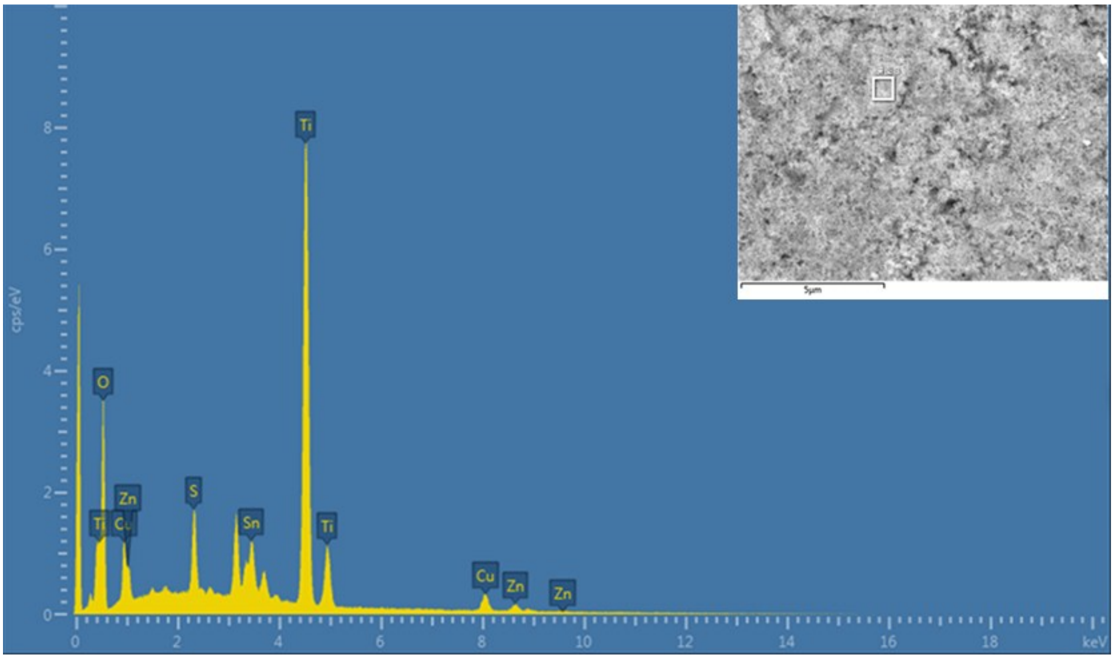

Fig.2S EDAS of the as-obtained CZTS prepared from precursors in DMF:Ethanol=1:1 solvent.

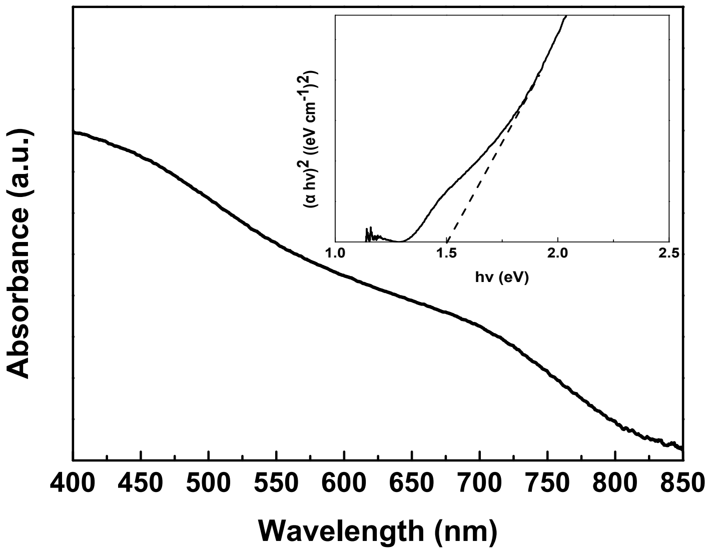

Fig.3S Absorption spectrum of the as-obtained CZTS prepared from precursors in DMF:Ethanol=1:1 solvent.

The absorption spectrum of the sulfurized CZTS prepared from precursors at a sintering temperature of 550°C 1h was characterized, revealing a broad absorption in visible region. The bandgap of CZTS is estimated to be 1.5 eV by plotting $(a\text{h}v)^2$-hv (where a = absorbance, h = Planck constant and v = frequency), as shown in inset of figure. The band gap energy is estimated by extrapolating the linear, this value is quite close to the theoretical optimal value required for a solar cell.

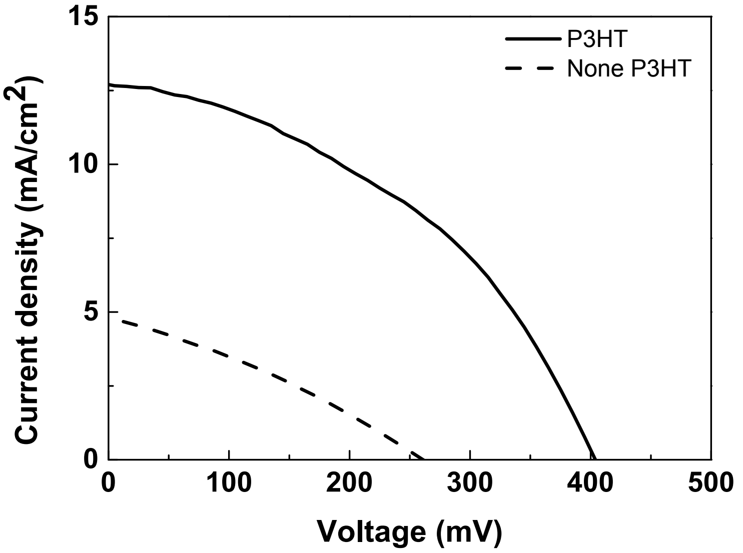

Fig. 4S J-V curves of the superstrate CZTS solar cells with or without hole transfer material.

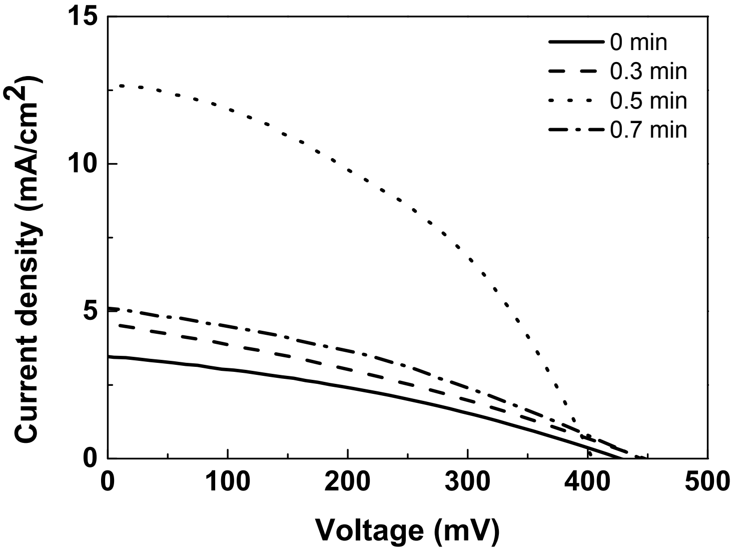

Fig.5S J-V curves of the superstrate CZTS solar cells based on ZnO modification layer electrodeposited for different times.
